# Quadrivalent Human Papillomavirus Vaccine and High-Grade Vulvovaginal Lesions

**DOI:** 10.1001/jamaoncol.2025.5511

**Published:** 2025-12-18

**Authors:** Yunyang Deng, Shiqiang Wu, Lina Schollin Ask, Tiia Lepp, Mark Clements, Hanna Milerad, Christina Carlander, Jiayao Lei

**Affiliations:** 1 Department of Medical Epidemiology and Biostatistics, Karolinska Institutet, Stockholm, Sweden; 2 Unit of Epidemiology, Institute of Environmental Medicine, Karolinska Institutet, Stockholm, Sweden; 3 Unit for Vaccination Programmes, Department of Public Health Analysis and Data Management, Public Health Agency of Sweden, Solna, Sweden; 4 Department of Women’s and Children’s Health, Karolinska Institutet, Stockholm, Sweden; 5 Sachs’ Children and Youth Hospital, Stockholm, Sweden; 6 Unit for Immunization, Department of Communicable Disease Control and Health Protection, Public Health Agency of Sweden, Solna, Sweden; 7 Regional Cancer Center - Stockholm and Gotland Region, Stockholm, Sweden; 8 Department of Clinical Science, Intervention and Technology, Karolinska Institutet, Stockholm, Sweden; 9 Department of Infectious Diseases, Karolinska University Hospital, Huddinge, Sweden; 10 Department of Medicine Huddinge, Karolinska Institutet, Huddinge, Sweden

## Abstract

**Importance:**

Human papillomavirus (HPV) vaccination has been associated with reduced risk of high-grade cervical lesions. However, evidence on its association with high-grade vulvovaginal lesions remains scarce.

**Objective:**

To evaluate the association between quadrivalent HPV vaccination and high-grade vulvovaginal lesions and assess the population-level incidence reduction among birth cohorts eligible for various vaccination programs.

**Design, Setting, and Participants:**

This population-based cohort study included women who were born between 1985 and 1998 and resided in Sweden between 2006 and 2022. Eligible participants had not previously received an HPV vaccination and did not have high-grade vulvovaginal lesions. Data were analyzed from February to October 2025.

**Exposure:**

HPV vaccination status was treated as a time-varying exposure, and birth cohorts corresponded to different vaccination programs: 1985 to 1988 (opportunistic vaccination program), 1989 to 1992 (subsidized vaccination), and 1993 to 1998 (catch-up vaccination).

**Main Outcome and Measures:**

The main outcome was incidence of high-grade vulvovaginal lesions, including cancers. Poisson regression models were used to estimate incidence rate ratios with 95% CIs.

**Results:**

Among 778 943 women, a total of 256 353 (32.9%) received at least 1 dose of the quadrivalent HPV vaccine. The median (IQR) follow-up duration was 17.0 (17.0-17.0) years for unvaccinated women, 12.2 (10.6-13.4) years for those vaccinated between ages 10 and 16 years, and 10.8 (9.3-13.5) years for those vaccinated at 17 years or older. During follow-up, 98 cases of high-grade vulvovaginal lesions were found in vaccinated women, and 547 cases were found in unvaccinated women. Compared with unvaccinated women, the fully adjusted incidence rate ratio of high-grade vulvovaginal lesions was 0.63 (95% CI, 0.50-0.81) in vaccinated women. Stratified by age at vaccination, the incidence rate ratios for those vaccinated at 10 to 16 years and 17 years or older were 0.45 (95% CI, 0.32-0.65) and 0.80 (95% CI, 0.61-1.06), respectively. Compared with women born between 1985 and 1988, the incidence rate ratios for those born in 1989 to 1992 and 1993 to 1998 were 0.81 (95% CI, 0.67-0.97) and 0.62 (95% CI, 0.49-0.80), respectively.

**Conclusions and Relevance:**

In this cohort study, vaccinated women had a lower incidence of high-grade vulvovaginal lesions compared with unvaccinated women, with a greater incidence reduction for those vaccinated at younger ages (before 17 years of age). Population-level incidence reduction was observed in cohorts vaccinated through subsidized or catch-up programs. These findings support that scaling up coverage of HPV vaccination at younger ages may help prevent high-grade vulvovaginal lesions.

## Key Points

**Question:**

What is the association between quadrivalent human papillomavirus (HPV) vaccination and high-grade vulvovaginal lesions?

**Findings:**

In this cohort study of 778 943 women, vaccinated women had a significantly lower incidence of high-grade vulvovaginal lesions than unvaccinated women, with a greater reduction among those vaccinated at ages 10 to 16 years than 17 years or older. A population-level incidence reduction was observed in birth cohorts covered by subsidized or catch-up vaccination programs compared with cohorts vaccinated opportunistically.

**Meaning:**

In this study, quadrivalent HPV vaccination was associated with reduced risk of high-grade vulvovaginal lesions, suggesting that expanding vaccination, especially at younger ages, could help prevent high-grade vulvovaginal lesions.

## Teaser

This cohort study evaluates the association between quadrivalent human papillomavirus vaccination and high-grade vulvovaginal lesions among women in Sweden.

## Introduction

Human papillomavirus (HPV) is the most common sexually transmitted infection, with an estimated lifetime infection risk exceeding 80% among sexually active individuals.^1^ A worldwide study found that HPV was responsible for 4.5% of all cancers, nearly 100% of cervical cancers, 24.9% of vulvar cancers, and 78.0% of vaginal cancers.^2^

In Sweden, HPV vaccination was introduced to women in 2006, with various delivery modes depending on administrative settings and personnel costs.^3,4^ Before May 2007, an opportunistic program was used, whereby vaccination was self-initiated and fully paid by individuals without any subsidy.^3,4^ In May 2007, a subsidized program was launched, targeting girls aged 13 to 17 years, with government support covering approximately 50% of the vaccine cost.^3,4^ From January 2012, a free-of-charge catch-up program was introduced for girls born between 1993 and 1998, alongside a school-based vaccination program at no cost for girls born in 1999 or later.^3,4^ The HPV vaccination coverage progressively increased from less than 10% under the initial opportunistic program to 35% following the subsidized program, with 55% through the catch-up program, and nearly 90% in school-based cohorts.^3,4^ Since its introduction in Sweden, the quadrivalent HPV vaccine (targeting HPV 6, 11, 16, and 18) has been mainly used, with the 9-valent HPV vaccine (HPV 6, 11, 16, 18, 31, 33, 45, 52, and 58) replacing it in October 2019.^3^

With the demonstrated effectiveness of HPV vaccination against HPV infections and cervical lesions,^5,6^ it is also expected to provide protection against noncervical HPV-associated malignant neoplasms. However, studies assessing its association with high-grade lesions or cancers of the vulva and vagina remain limited. A systematic review of phase II and III trials showed that HPV vaccination was associated with a 71% reduced risk of vulvar and vaginal precancers and cancers in an intention-to-treat analysis.^7^ Additionally, only 1 observational study from Denmark reported those who received the HPV vaccine had a 52% lower risk of high-grade vulvar lesions and a 70% reduced risk of high-grade vaginal lesions.^8^ The risk reduction was more pronounced among women vaccinated before 17 years of age.^8^

In this population-based cohort study, we aimed to assess the association between quadrivalent HPV vaccination and high-grade vulvovaginal lesions using data from nationwide Swedish registries. We also measured the population-level incidence difference across birth cohorts eligible for various HPV vaccination programs.

## Methods

### Data Source and Study Population

This cohort study was approved by the regional ethical review board in Stockholm. The review board waived the need for written informed consent in accordance with Swedish law, as the study was based on pseudonymized registry data collected for public health purposes, where obtaining individual consent is impracticable, the risk to participants is minimal, and strict legal safeguards protect individual privacy. We followed the Strengthening the Reporting of Observational Studies in Epidemiology (STROBE) reporting guideline. This study used nationwide Swedish population and health care registers linked at the individual level.^9^ HPV vaccination data were sourced from the Swedish HPV Vaccination Register (SVEVAC),^3^ National Vaccination Register (NVR),^10^ and Prescribed Drug Register (PDR).^11^ Information on precancers and cancers was obtained from the National Patient Register (NPR)^12^ and National Cancer Register (NCR).^13^ Immigration, emigration, birth year, birth country, and residence county were collected from the Total Population Register.^14^ Death records were retrieved from the Cause of Death Register.^15^ Education and income were obtained from the Longitudinal Integration Database for Health Insurance and Labor Market Studies.^16^ Womens’ parents were linked via the Multi-Generation Register.^17^

**Figure 1.**
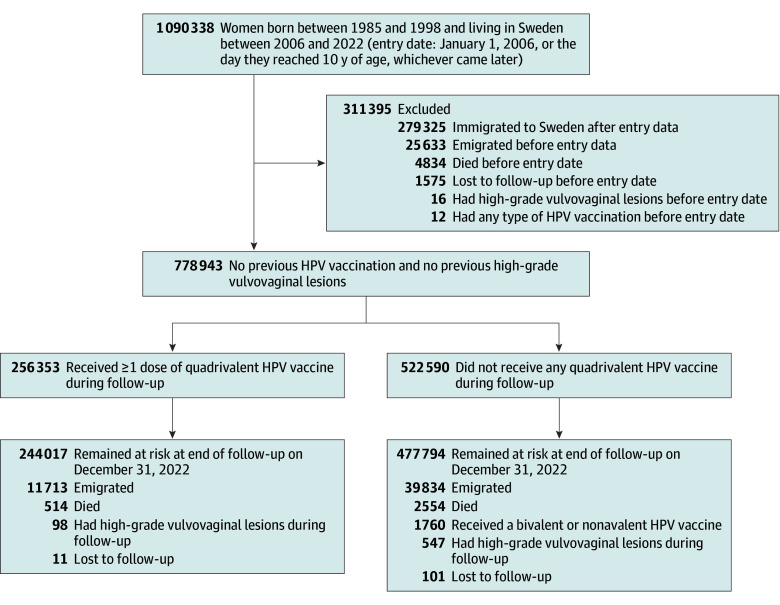
Flowchart of the Study Population HPV indicates human papillomavirus.

From registers, we identified women born between 1985 and 1998 living in Sweden during 2006 through 2022, and followed up all women from January 1, 2006, or the date they reached 10 years of age, whichever occurred later (the entry date). We excluded those who immigrated to Sweden after the entry date, as their HPV vaccination records before immigration were unclear. Additionally, we excluded women who died, emigrated, were lost to follow-up, had any HPV vaccination, or had high-grade vulvovaginal lesions before the entry date. All women were followed up until they developed the outcome, died, emigrated, were lost to follow-up, received bivalent or nonavalent HPV vaccinations (given that the exposure was quadrivalent vaccination), or until follow-up ended (December 31, 2022), whichever occurred first (Figure 1).

### HPV Vaccination

Women who had at least 1 dose of quadrivalent HPV vaccine during follow-up were considered as vaccinated. Vaccination status was considered as a time-varying exposure in this study. The SVEVAC is a voluntary registry that recorded HPV vaccinations from 2006 to 2015.^3^ The NVR has documented virtually complete vaccination data for the national vaccination program since 2013.^10^ The PDR, established in 2005, has recorded all prescribed drugs that are dispensed, including subsidized HPV vaccines.^11^ In this study, we used the SVEVAC and NVR as primary sources, supplementing with data from the PDR.

### High-Grade Vulvovaginal Lesions

The outcome was defined as the first diagnosis of high-grade vulvovaginal lesions during follow-up, including high-grade vulvar lesions and invasive vulvar cancer, as well as high-grade vaginal lesions and invasive vaginal cancer. The NPR provides virtually complete data on all inpatient care since 1987 and outpatient specialized care since 2001.^12^ The NCR, established in 1958, is a mandatory nationwide cancer registry with near-complete coverage.^13^ In this study, vulvovaginal precancers were sourced from both NPR and NCR, whereas vulvovaginal cancers were only obtained from NCR to ensure the accuracy of cancer cases. The identification was based on the *International Statistical Classification of Diseases and Related Health Problems, Tenth Revision* codes for the vulva (N901, N902, D071, C51) and vagina (N891, N892, D072, C52). The index date of first diagnosis was defined as the first admission date from the NPR, or the date of diagnosis from the NCR.

### Covariates

Covariates included attained age, calendar year, county of residence, mother’s birth country, highest parental education level, annual household income, and parental history of HPV-related precancers and cancers (high-grade vulvar lesions, high-grade vaginal lesions, cervical intraepithelial neoplasia grade 3 or worse, high-grade anal lesions, oropharyngeal cancer, and penile cancer). Education was classified into 3 categories based on educational attainment: low (less than high school), medium (high school), and high (equivalent to university or above).^5^ Income was categorized into low, medium, and high, determined by the income tertiles of the population aged 20 to 65 years.^5^ For any covariate with missing values, we introduced an extra “missing” category.^5^ All covariates except attained age, calendar year, and mother’s birth country were measured in the year before study entry.

### Statistical Analysis

Data were analyzed from February to October 2025. Baseline characteristics were shown based on HPV vaccination status and age at first vaccination, which was categorized as 10 to 16 years and 17 years or later according to the median age of sexual debut for women in Sweden (17 years)^18^ and our previous cutoff.^5,6^ The cumulative incidence of high-grade vulvovaginal lesions was plotted according to vaccination status and attained age using the Kaplan-Meier method. Vaccination status was considered a time-varying exposure, allowing the same individual to contribute person-time to both unvaccinated and vaccinated groups. An individual was moved from the unvaccinated group to the vaccinated group on the date of the first quadrivalent HPV vaccine. We used attained age as the underlying time scale, with follow-up beginning at each individual’s age at study entry (delayed entry). Each individual was at risk for high-grade vulvovaginal lesions from the age at start of follow-up until the age at end of follow-up.

Poisson regression models were used to estimate incidence rate ratios (IRRs) with 95% CIs comparing vaccinated and unvaccinated women and different ages at vaccination. Attained age and calendar year were included as time-varying variables by splitting follow-up time into shorter intervals for each woman, corresponding to 1-year changes in age and progression in calendar time simultaneously. We calculated the calendar year by taking the sum of birth year and attained age during follow-up. Two models were generated: (1) an age-adjusted model, adjusted for attained age as a natural spline term with 3 degrees of freedom; and (2) a fully adjusted model, further adjusted for calendar year as a categorical variable with 1-year levels, county of residence, mother’s birth country, highest parental education level, annual household income level, and parental history of HPV-related precancers and cancers.

Additionally, we divided the birth cohorts into 3 groups: 1985 to 1988 (opportunistic cohorts), 1989 to 1992 (subsidized cohorts), and 1993 to 1998 (catch-up cohorts). Using the birth group from 1985 to 1988 as the reference, we estimated IRRs and 95% CIs for cohorts’ vaccination through subsidized and catch-up programs using Poisson regression models, adjusting for the same set of covariates as specified previously except for calendar year.

In sensitivity analyses, we introduced 1- and 2-year buffer periods between vaccination and case-counting to exclude prevalent HPV infections at the time of vaccination. The person-time for vaccinated women during this buffer period was included in the unvaccinated group. Additionally, we analyzed the association between HPV vaccination and high-grade vulvar and vaginal lesions separately.

Statistical significance was defined as a 2-sided *P* < .05. Data management was performed in SAS 9.4 and data analysis was conducted with Stata, version 18 (StataCorp LLC).

## Results

### Study Population and Follow-Up

The study included 778 943 women who were born between 1985 and 1998 and resided in Sweden during 2006 to 2022. Of these women, 256 353 (32.9%) received at least 1 dose of quadrivalent HPV vaccination. During follow-up, 98 and 547 incident cases of high-grade vulvovaginal lesions were identified in vaccinated and unvaccinated groups, respectively (Figure 1). The median follow-up duration was 17.0 years (IQR, 17.0-17.0 years) for unvaccinated women, 12.2 years (IQR, 10.6-13.4 years) for those vaccinated at 10 to 16 years, and 10.8 years (IQR, 9.3-13.5 years) for those vaccinated at 17 years or older. The median age at first vaccination was 15 years (IQR, 10-16 years) for those vaccinated at 10 to 16 years and 18 years (IQR, 17-21 years) for those vaccinated at 17 years or older (Table 1).

**Table 1.** Characteristics of the Study Population at Baseline

Variable	Unvaccinated	Vaccinated at age 10-16 y^a^	Vaccinated at age ≥17 y^a^
Total population, No. (%)^b^	522 590 (67.1)	163 208 (21.0)	93 145 (12.0)
Age at first vaccination, median (IQR), y	NA	15 (10-16)	18 (17-21)
Follow-up, median (IQR), y^c^	17.0 (17.0-17.0)	12.2 (10.6-13.4)	10.8 (9.3-13.5)
Birth cohort, No. (%)^b^^,^^d^			
1985-1988	206 325 (94.8)	0	14 972 (5.2)
1989-1992	180 754 (76.8)	25 467 (9.7)	41 803 (13.5)
1993-1998	135 511 (56.8)	137 741 (35.2)	36 370 (8.0)
County of residence, No. (%)^d^^,^^e^			
Stockholm, Sweden	92 089 (17.6)	36 370 (22.3)	23 973 (25.7)
Other counties	417 809 (79.9)	126 461 (77.5)	69 112 (74.2)
Missing data^f^	12 692 (2.4)	377 (0.2)	60 (0.1)
Mother’s country of birth, No. (%)^d^			
Sweden	401 381 (76.8)	143 758 (88.1)	78 773 (84.6)
Other countries	105 568 (20.2)	19 189 (11.8)	14 152 (15.2)
Missing data^f^	15 641 (3.0)	261 (0.2)	220 (0.2)
Highest parental education level, No. (%)^d^^,^^g^			
Low	33 892 (6.5)	3412 (2.1)	3075 (3.3)
Middle	259 838 (49.7)	63 939 (39.2)	38 171 (41.0)
High	212 054 (40.6)	95 310 (58.4)	51 639 (55.4)
Missing data^f^	16 806 (3.2)	547 (0.3)	260 (0.3)
Annual household income level, No. (%)^d^^,^^h^			
Low	56 057 (10.7)	11 601 (7.1)	7029 (7.5)
Middle	204 421 (39.1)	55 512 (34.0)	31 845 (34.2)
High	245 780 (47.0)	95 559 (58.6)	53 967 (57.9)
Missing data^f^	16 332 (3.1)	536 (0.3)	304 (0.3)
Parental history of HPV-related precancers and cancers, No. (%)^f^^,^^i^			
No	501 168 (95.9)	156 640 (96.0)	89 012 (95.6)
Yes	21 422 (4.1)	6568 (4.0)	4133 (4.4)

Abbreviations: NA, not applicable; HPV, human papillomavirus.

^a^ Received at least 1 dose of quadrivalent HPV vaccination.

^b^ Row percentage.

^c^ Total follow-up time was defined as the period from entry of study to end of study for unvaccinated women, and from the first vaccine dose to end of study for vaccinated women.

^d^ Percentages may not total 100 because of rounding.

^e^ County of residence was adjusted in Poisson models based on each county.

^f^ Overall, 97.07% of women do not have any missing data.

^g^ The highest parental education level was classified as low if the parent had 9 years or less of primary education, middle if the parent had 2 to 3 years of secondary schooling (similar to senior high school), and high if the parent had postsecondary education and above (equivalent to university studies).

^h^ Annual household income level was categorized into low, medium, and high based on the income level tertiles of the population aged 20 to 65 years.

^i^ HPV-related precancers and cancers included high-grade vulvar lesions, high-grade vaginal lesions, cervical intraepithelial neoplasia grade 3 or worse, high-grade anal lesions, oropharyngeal cancer, and penile cancer.

### Incidence of High-Grade Vulvovaginal Lesions by HPV Vaccination Status

**Figure 2.**
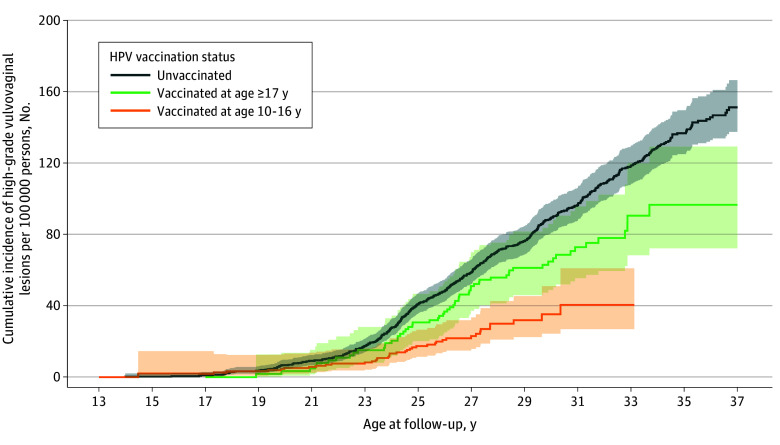
Cumulative Incidence of High-Grade Vulvovaginal Lesions Stratified by Human Papillomavirus (HPV) Vaccination Status and Age at First Vaccination HPV vaccination was treated as a time-varying exposure. Attained age was used as the underlying time scale, with follow-up beginning at each individual’s age at study entry (delayed entry). For example, an individual vaccinated at age 20 years contributes person-time to the unvaccinated curve from cohort entry until age 20 years, and then to the vaccinated curve from age 20 years onward. If this individual had developed the outcome before age 20 years, the event would have been counted in the unvaccinated group. Age at follow-up was truncated in the graph because no cases of high-grade vulvovaginal lesions were observed in girls younger than 13 years of age. The shaded areas represent 95% CIs.

The cumulative incidence of high-grade vulvovaginal lesions began to increase markedly at 23 years of age across all groups (Figure 2). The unvaccinated group exhibited the highest incidence, with a consistent upward trend over time. Those vaccinated at 10 to 16 years had the lowest incidence, while women vaccinated at 17 years or older showed an intermediate incidence (Figure 2). The crude incidence rates per 100 000 person-years were 3.28 (95% CI, 2.69-4.00) in those vaccinated and 5.72 (95% CI, 5.26-6.22) among those unvaccinated, with age-adjusted and fully adjusted IRRs of 0.55 (95% CI, 0.44-0.68) and 0.63 (95% CI, 0.50-0.81), respectively (Table 2). Stratified by age at vaccination, the crude incidence rates per 100 000 person-years were 1.98 (95% CI, 1.45-2.71) for those vaccinated at 10 to 16 years and 5.79 (95% CI, 4.48-7.47) for those aged 17 years or older. The fully adjusted IRRs for those vaccinated at 10 to 16 years and 17 years or older compared with unvaccinated women were 0.45 (95% CI, 0.32-0.65) and 0.80 (95% CI, 0.61-1.06), respectively (Table 2).

**Table 2.** Incidence Rate Ratios of High-Grade Vulvovaginal Lesions by HPV Vaccination

HPV vaccination status	Person-years	No. of cases	Crude incidence rate per 100 000 person-years (95% CI)	Incidence rate ratio (95% CI)	
				Age-adjusted^a^	Fully adjusted^b^
Unvaccinated	9 559 727	547	5.72 (5.26-6.22)	1 [Reference]	1 [Reference]
Vaccinated	2 988 564	98	3.28 (2.69-4.00)	0.55 (0.44-0.68)	0.63 (0.50-0.81)
Age at first vaccination, y					
10-16	1 968 708	39	1.98 (1.45-2.71)	0.39 (0.28-0.55)	0.45 (0.32-0.65)
≥17	1 019 856	59	5.79 (4.48-7.47)	0.74 (0.56-0.97)	0.80 (0.61-1.06)

Abbreviation: HPV, human papillomavirus.

^a^ Adjusted for age as a spline with 3 degrees of freedom.

^b^ Adjusted for age as a spline term with 3 degrees of freedom, calendar year, county of residence, mother’s country of birth, highest parental education level, annual household income level, and parental history of HPV-related precancers and cancers.

### Incidence of High-Grade Vulvovaginal Lesions by Birth Cohorts

The crude incidence rates per 100 000 person-years were 8.90 (95% CI, 7.97-9.94) for women born from 1985 to 1988, 5.01 (95% CI, 4.36-5.75) for those born from 1989 to 1992, and 2.53 (95% CI, 2.13-3.02) for women born from 1993 to 1998. The fully adjusted IRRs were 0.81 (95% CI, 0.67-0.97) for the 1989 to 1992 cohort and 0.62 (95% CI, 0.49-0.80) for the 1993 to 1998 cohort (Table 3).

**Table 3.** Incidence Rate Ratios of High-Grade Vulvovaginal Lesions by Birth Cohorts Eligible for Various HPV Vaccination Programs

Birth cohort (vaccination program and coverage) [vaccination coverage]	No. of total population	Person-years	No. of cases	Crude incidence rate per 100 000 person-years (95% CI)	Incidence rate ratio (95% CI)	
					Age-adjusted	Fully adjusted^a^
1985-1988 (Opportunistic cohorts) [6.77%]	221 297	35 614 789	317	8.90 (7.97-9.94)	1 [Reference]	1 [Reference]
1989-1992 (Subsidized cohorts) [27.12%]	248 024	4 051 714	203	5.01 (4.37-5.75)	0.81 (0.67-0.98)	0.81 (0.67-0.97)
1993-1998 (Catch-up cohorts) [56.23%]	309 622	4 935 098	125	2.53 (2.13-3.02)	0.62 (0.48-0.79)	0.62 (0.49-0.80)

Abbreviation: HPV, human papillomavirus.

^a^ Adjusted for age, county of residence, mother’s country of birth, highest parental education level, annual household income level, and parental history of HPV-related precancers and cancers.

### Sensitivity Analysis

When a 1-year buffer period was applied, the fully adjusted IRR for vaccinated women was 0.57 (95% CI, 0.45-0.74), with IRRs of 0.43 (95% CI, 0.30-0.62) and 0.71 (95% CI, 0.53-0.96) for women who had been vaccinated at ages 10 to 16 years and 17 years or older, respectively (eTable 1 in Supplement 1). Similar findings were observed when using a 2-year buffer period (eTable 1 in Supplement 1).

When stratifying the outcome by anatomic site, the fully adjusted IRRs for overall vaccination and vaccination at ages 10 to 16 years and 17 years or older compared with unvaccinated women were 0.63 (95% CI, 0.44-0.89), 0.43 (95% CI, 0.25-0.72), and 0.82 (95% CI, 0.55-1.22), respectively, for high-grade vulvar lesions (eTable 2 in Supplement 1). Similar results were observed for high-grade vaginal lesions, with fully adjusted IRRs of 0.63 (95% CI, 0.46-0.87), 0.47 (95% CI, 0.29-0.75), and 0.78 (95% CI, 0.54-1.13), respectively (eTable 2 in Supplement 1).

## Discussion

This population-based cohort study showed that women who received at least 1 dose of the quadrivalent HPV vaccine had a lower incidence of high-grade vulvovaginal lesions than those unvaccinated. Similar results were observed when examining vulvar and vaginal lesions separately. The incidence reduction of high-grade vulvovaginal lesions was statistically significant among women vaccinated at 10 to 16 years. We observed a reduced incidence for those vaccinated at 17 years or older, though the difference was not statistically significant. However, when applying a buffer period of 1 or 2 years, the incidence of high-grade vulvovaginal lesions was significantly lower among those vaccinated at 17 years or older. Additionally, this study showed that birth cohorts eligible for subsidized and catch-up programs exhibited a lower incidence of high-grade vulvovaginal lesions than birth cohorts vaccinated opportunistically.

Consistent with previous research, our results showed that receiving HPV vaccination was associated with reduced risk of high-grade vulvovaginal lesions. A systematic review of trials reported a 71% lower risk of vulvar and vaginal precancers and cancers for those with HPV vaccination.^7^ A cohort study from Denmark showed that HPV-vaccinated women had a 52% lower risk of high-grade vulvar lesions and a 70% reduced risk of high-grade vaginal lesions than unvaccinated women.^8^ Similarly in the present study, the incidence reduction was statistically significant exclusively in those vaccinated before 17 years of age.^8^ These findings emphasize the critical role of HPV vaccination initiated at an early age. The enhanced risk reduction in younger women may be attributable to the reduced probability of prior HPV exposure before vaccination.^19^ Though the risk reduction for those vaccinated at 17 years or older did not reach a statistical difference, when applying buffer periods to exclude the risk of prevalent HPV infections at the time of vaccination,^5^ we did observe significant risk reductions for those vaccinated at 17 years or older. Therefore, we think that HPV vaccination at an older age was also associated with reduced risk of high-grade vulvovaginal lesions.

Our findings demonstrate that women in the subsidized (1989-1992) and catch-up (1993-1998) birth cohorts exhibited a lower incidence of high-grade vulvovaginal lesions than those in the opportunistic (1985-1988) birth cohort. The differences likely reflect variations in HPV vaccine coverage and the age at vaccination across different programs. The opportunistic cohort had access to the HPV vaccine primarily through private health care, leading to lower and more socioeconomically selective coverage.^4^ In contrast, the subsidized cohort benefited from a partially funded vaccination program, which likely improved accessibility and coverage.^4^ The catch-up cohort experienced a higher coverage due to the introduction of a national vaccination program, which provided vaccination at no cost to targeted age groups.^4^ Additionally, women in the catch-up cohort were generally vaccinated at a younger age, before potential HPV exposure, which is crucial for maximizing vaccine effectiveness. The greater risk reduction observed in the catch-up cohort highlights the importance of organized, publicly funded vaccination programs in achieving high coverage and ensuring vaccination at an optimal age. These results support continued approaches to enhance HPV vaccination uptake, particularly through school-based and publicly funded initiatives, to maximize population-level protection against HPV-associated diseases.

Given that our study is population-based and encompasses women from diverse demographic and socioeconomic backgrounds, our findings are likely generalizable to countries with well-established HPV vaccination programs and comparable coverage levels. Future research could investigate the effectiveness of different types of HPV vaccines, particularly vaccines that cover a broader range of HPVs and the effectiveness of varying vaccination dose regimens. Further studies could also assess the vaccine’s effectiveness against vulvovaginal cancer, which was not examined separately from high-grade vulvovaginal lesions in this study due to the limited number of cancer cases. Meanwhile, with the implementation of the sex-neutral vaccination program, future studies are encouraged to assess the effectiveness of HPV vaccination against other HPV-related precancers and cancers, including not only those occurring in women but also those occurring in men, such as penile, anal, and oropharyngeal precancers and cancers.

### Strengths and Limitations

Our study has some strengths. First, this population-based cohort study leveraged Swedish nationwide registries, ensuring valid and reliable data while providing sufficient statistical power to estimate age-specific associations within real-world vaccination programs. Second, we conducted a sensitivity analysis using buffer periods to exclude prevalent HPV infections, which might otherwise lead to an overestimation of incidence in the vaccinated group.^5^ Third, we assessed the association between quadrivalent HPV vaccination and high-grade vulvovaginal lesions across different birth cohorts corresponding to various HPV vaccination programs. Lastly, we incorporated comprehensive confounders.

Nevertheless, there are several limitations. First, there is a potential for misclassification of vaccination status. A small proportion of vaccinated women (8% of all doses administered in 2006-2015) were misclassified as unvaccinated due to anonymous records in SVEVAC resulting from lack of informed consent.^6^ However, these misclassifications are expected to bias the estimates toward the null. Second, the absence of a systematic screening program for vulvovaginal diseases may cause some outcomes to remain undiagnosed, leading to an underestimation of the incidence for both comparison groups. However, the current data represent the most comprehensive data available from registers. If anything, the nondifferential misclassification would bias our estimates toward the null. Third, certain high-grade vulvovaginal lesions, particularly asymptomatic or mild cases, could be detected through cervical screening or gynecological examinations. Vaccinated women may be more likely to attend screening, potentially leading to higher detection rates, which would only result in an underestimation of the observed association. Fourth, the well-known challenge of distinguishing between cervical and vaginal lesion origins^8^ means that some lesions of cervical origin might be misclassified as vaginal. Fifth, HPV status of high-grade vulvovaginal lesions was unavailable. Unlike cervical cancer, not all vulvovaginal cancers—particularly those of the vulva—are attributable to HPV. Nevertheless, the proportion of HPV-negative cases among high-grade vulvovaginal lesions is relatively low (5%-15%).^20^ Sixth, vaccinated women might practice overall healthier behaviors than unvaccinated women, potentially resulting in an overestimation of the risk reduction. To address this possibility, we adjusted for comprehensive covariates. Nevertheless, we cannot completely rule out the possibility of residual confounding due to unaccounted-for factors, such as smoking and sexual activity, as these data are unavailable in registers. Instead, we adjusted for parental education and household income, which may serve as partial proxies for some factors.^21^ Lastly, our results have limitations in terms of causal interpretation due to the observational design and the fact that individuals vaccinated at 17 years or older should be event-free before vaccination. However, we noted that there were 4 outcomes before age 17 years in the unvaccinated group, 1 in those vaccinated at 10 to 16 years, and 0 in those vaccinated at 17 years or older, before treating HPV vaccination as time-varying.

## Conclusion

In conclusion, this population-based cohort study found that women who received at least 1 dose of the quadrivalent HPV vaccine had a lower incidence of high-grade vulvovaginal lesions than unvaccinated women. The incidence reduction was more pronounced among those vaccinated at age 10 to 16 years. A population-level reduction in high-grade vulvovaginal lesions was observed among birth cohorts vaccinated through subsidized or catch-up programs, highlighting the significance of early and widespread vaccination in lowering the burden of these lesions.

## Notes

Supplement 1. **eTable 1.** Incidence rate ratios of high-grade vulvovaginal lesions by HPV vaccination, with 1 or 2 years buffer period **eTable 2.** Incidence rate ratios for high-grade vulvar lesions and vaginal lesions by HPV vaccination

Supplement 2.
Data Sharing Statement
