# Supplementary material for: Quadrivalent Human Papillomavirus Vaccine and High-Grade Vulvovaginal Lesions
Source: JAMA Oncol. 2025 Dec 18;12(2):177–84. doi: 10.1001/jamaoncol.2025.5511 (PMC12921526; doi:10.1001/jamaoncol.2025.5511)
Supplement: Supplement 1. — eTable 1. Incidence rate ratios of high-grade vulvovaginal lesions by HPV vaccination, with 1 or 2 years buffer period eTable 2. Incidence rate ratios for high-grade vulvar lesions and vaginal lesions by HPV vaccination [file jamaoncol-e255511-s001.pdf]

Supplemental Online Content

Deng Y, Wu S, Ask LS, et al. Quadrivalent human papillomavirus vaccine and high-grade vulvovaginal lesions. *JAMA Oncol*. Published online December 18, 2025. doi:10.1001/jamaoncol.2025.5511

eTable 1. Incidence rate ratios of high-grade vulvovaginal lesions by HPV vaccination, with 1 or 2 years buffer period

eTable 2. Incidence rate ratios for high-grade vulvar lesions and vaginal lesions by HPV vaccination

This supplemental material has been provided by the authors to give readers additional information about their work.

© 2025 Deng Y et al. JAMA Oncology.

eTable 1. Incidence rate ratios of high-grade vulvovaginal lesions by HPV vaccination, with 1 or 2 years buffer period

Buffer period	HPV vaccination status	No. of cases	Age-adjusted incidence rate ratio [a] (95% CI)	Fully adjusted incidence rate ratio [b] (95% CI)
**No buffer period (main analysis)**	Unvaccinated	547	Reference	Reference
	Vaccinated	98	0.55 (0.44 - 0.68)	0.63 (0.50 - 0.81)
	Vaccinated at 10-16 years	39	0.39 (0.28 - 0.55)	0.45 (0.32 - 0.65)
	Vaccinated at 17+ years	59	0.74 (0.56 - 0.97)	0.80 (0.61 - 1.06)
**1-year**	Unvaccinated	556	Reference	Reference
	Vaccinated	89	0.51 (0.40 - 0.64)	0.57 (0.45 - 0.74)
	Vaccinated at 10-16 years	38	0.38 (0.28 - 0.53)	0.43 (0.30 - 0.62)
	Vaccinated at 17+ years	51	0.66 (0.50 - 0.88)	0.71 (0.53 - 0.96)
**2-year**	Unvaccinated	565	Reference	Reference
	Vaccinated	80	0.47 (0.37 - 0.59)	0.52 (0.40 - 0.67)
	Vaccinated at 10-16 years	36	0.37 (0.26 - 0.51)	0.41 (0.28 - 0.59)
	Vaccinated at 17+ years	44	0.60 (0.44 - 0.81)	0.64 (0.47 - 0.88)

HPV, human papillomavirus; CI, confidence interval.
[a] Adjusted for age as a spline with 3 degrees of freedom.
[b] Adjusted for age as a spline term with 3 degrees of freedom, calendar year, county of residence, mother's country of birth, highest parental education level, annual household income level, and parental history of HPV-related precancers/cancers.

© 2025 Deng Y et al. JAMA Oncology.

eTable 2. Incidence rate ratios for high-grade vulvar lesions and vaginal lesions by HPV vaccination

HPV vaccination status	Person-years (py) [a]	No. of cases [a]	Crude incidence rate per 100,000 py (95% CI)	Age-adjusted incidence rate ratio [b] (95% CI)	Fully adjusted incidence rate ratio [c] (95% CI)
**High-grade vulvar lesions**					
Unvaccinated	9,561,567	264	2.76 (2.45 - 3.12)	Reference	Reference
Vaccinated	2,988,843	47	1.57 (1.18 - 2.09)	0.55 (0.40 - 0.76)	0.63 (0.44 - 0.89)
Age at first vaccination					
10-16 years	1,968,773	18	0.92 (0.58 - 1.45)	0.37 (0.23 - 0.60)	0.43 (0.25 - 0.72)
17+ years	1,020,069	29	2.84 (1.98 - 4.09)	0.79 (0.54 - 1.16)	0.82 (0.55 - 1.22)
**High-grade vaginal lesions**					
Unvaccinated	9,561,305	315	3.30 (2.95 - 3.68)	Reference	Reference
Vaccinated	2,988,956	56	1.87 (1.44 - 2.44)	0.55 (0.41 - 0.73)	0.63 (0.46 - 0.87)
Age at first vaccination					
10-16 years	1,968,780	23	1.17 (0.78 - 1.76)	0.41 (0.27 - 0.64)	0.47 (0.29 - 0.75)
17+ years	1,020,176	33	3.24 (2.30 - 4.55)	0.70 (0.49 - 1.00)	0.78 (0.54 - 1.13)

HPV, human papillomavirus; CI, confidence interval.
[a] Total person-years differ among outcome types due to exclusion of the specific outcomes before start of follow-up.
[b] Adjusted for age as a spline with 3 degrees of freedom.
[c] Adjusted for age as a spline term with 3 degrees of freedom, calendar year, county of residence, mother's country of birth, highest parental education level, annual household income level, and parental history of HPV-related precancers/cancers.

© 2025 Deng Y et al. JAMA Oncology.
