# Supplementary material for: Quadrivalent Human Papillomavirus Vaccine and High-Grade Vulvovaginal Lesions
Source: JAMA Oncol. 2025 Dec 18;12(2):177–84. doi: 10.1001/jamaoncol.2025.5511 (PMC12921526; doi:10.1001/jamaoncol.2025.5511)
Supplement: Supplement 2. — Data Sharing Statement [file jamaoncol-e255511-s002.pdf]

Data Sharing Statement

Deng. Quadrivalent Human Papillomavirus Vaccine and High-Grade Vulvovaginal Lesions. *JAMA Oncol*. Published December 18, 2025. doi:10.1001/jamaoncol.2025.5511

Data
Data available: No

Additional Information
Explanation for why data not available: Data sharing statement: The raw datasets are not available for sharing because of privacy policies and regulations in Sweden. Additional data is available on request from the corresponding author for any interested researchers provided all ethical and legal requirements are met.
